# Femoral artery aneurysm with large hematoma from *Pasteurella*: case report and literature review

**DOI:** 10.1186/s12879-022-07136-5

**Published:** 2022-02-21

**Authors:** Jamie Hanson, Ali Etemady-Deylamy, Jared Frisby, Justin D’Addario, Matthew Smeds, Robin Chamberland, Huazhang Guo, Getahun Abate

**Affiliations:** 1 School of Medicine, Saint Louis University, Saint Louis, MO USA; 2 Department of Internal Medicine, Division of Infectious Diseases, Saint Louis University, Saint Louis, MO USA; 3 Department of Surgery, Division of Vascular and Endovascular Surgery, Saint Louis University, Saint Louis, MO USA; 4 SSM Saint Louis Network Microbiology, Saint Louis, MO USA; 5 Department of Pathology, Saint Louis University, Saint Louis, MO USA

**Keywords:** Case report, *Pasteurella*, Mycotic aneurysm, Femoral artery

## Abstract

**Background:**

*Pasteurella multocida* is a well-known gram-negative facultative anaerobe well known for its ability to cause soft tissue infections following animal bite or scratch. Here we present a case with mycotic aneurysm of the superficial femoral artery due to *P. multocida* infection.

**Case presentation:**

A 62 year old male patient presented with worsening right leg pain and swelling. On examination, he was found to have profound swelling and erythema of the right medial thigh and tenderness to palpation. Computerized tomography showed findings suggestive of right femoral pseudoaneurysm with a large right medial thigh hematoma. Blood cultures grew *P. multocida*. Patient underwent emergent open resection of the mycotic aneurysm and vascular bypass surgery. Intraoperatively, the site was noted to be grossly infected with multiple pockets of pus which were drained and pus cultures grew *P. multocida.* The diagnosis of *P. multocida* bacteremia with right femoral mycotic aneurysm and thigh abscess was made. Patient received 6 weeks of intravenous ceftriaxone and recovered.

**Conclusion:**

Our case is the first report on infection of peripheral vessel with *Pasteurella* and highlights the importance of prompt surgical intervention and effective antibiotic treatment

## Background

*Pasteurella multocida* (*P. multocida*) is a well-known gram-negative facultative anaerobic coccobacillus present in the normal oral flora of animals, with cats and dogs having the highest carriage rates. The bacteria typically are introduced to the body through animal bites, scratches, or oral secretions in licking. Infection with *P. multocida* commonly manifests as localized cellulitis, abscesses, tenosynovitis, osteomyelitis, and septic arthritis [1]. Disseminated infection is rare and more frequently impacts cirrhotic patients and those with additional immunocompromising comorbidities such as malignancy and renal disease [2]. In these patients, *P. multocida* infection can cause meningitis, peritonitis, and urogenital infections. Mycotic aneurysm caused by *P. multocida* is rare and only 10 reports of *P. multocida* mycotic aneurysm have been described to date. In this case report, we describe a 62-year-old male with a mycotic aneurysm of superficial femoral artery due to *P. multocida*. To the best of our knowledge, this is the first case of peripheral artery mycotic aneurysm caused by *P. multocida*.

## Case presentation

A 62-year-old man with a past medical history of treated hepatitis C, hepatitis B, cirrhosis, chronic obstructive pulmonary disease, chronic low back pain status post lumbar laminectomy and fusion presented to a local community hospital with progressively worsening right leg pain and swelling of 2–3 weeks duration. Physical examination revealed profound swelling and erythema of the right medial thigh and tenderness of right thigh to palpation. Social history was significant for a 40-pack year history of cigarette smoking, intravenous drug use and alcohol abuse. The last intravenous drug use was more than 5 years ago and he only used superficial veins of his left arm. Upon further questioning, the patient reported living at a friend’s home for the past 2.5 years with 15 dogs and 1 cat. He claims that he sustained numerous scratches on his bilateral arms and abdomen from the pets.

**Fig. 1:**
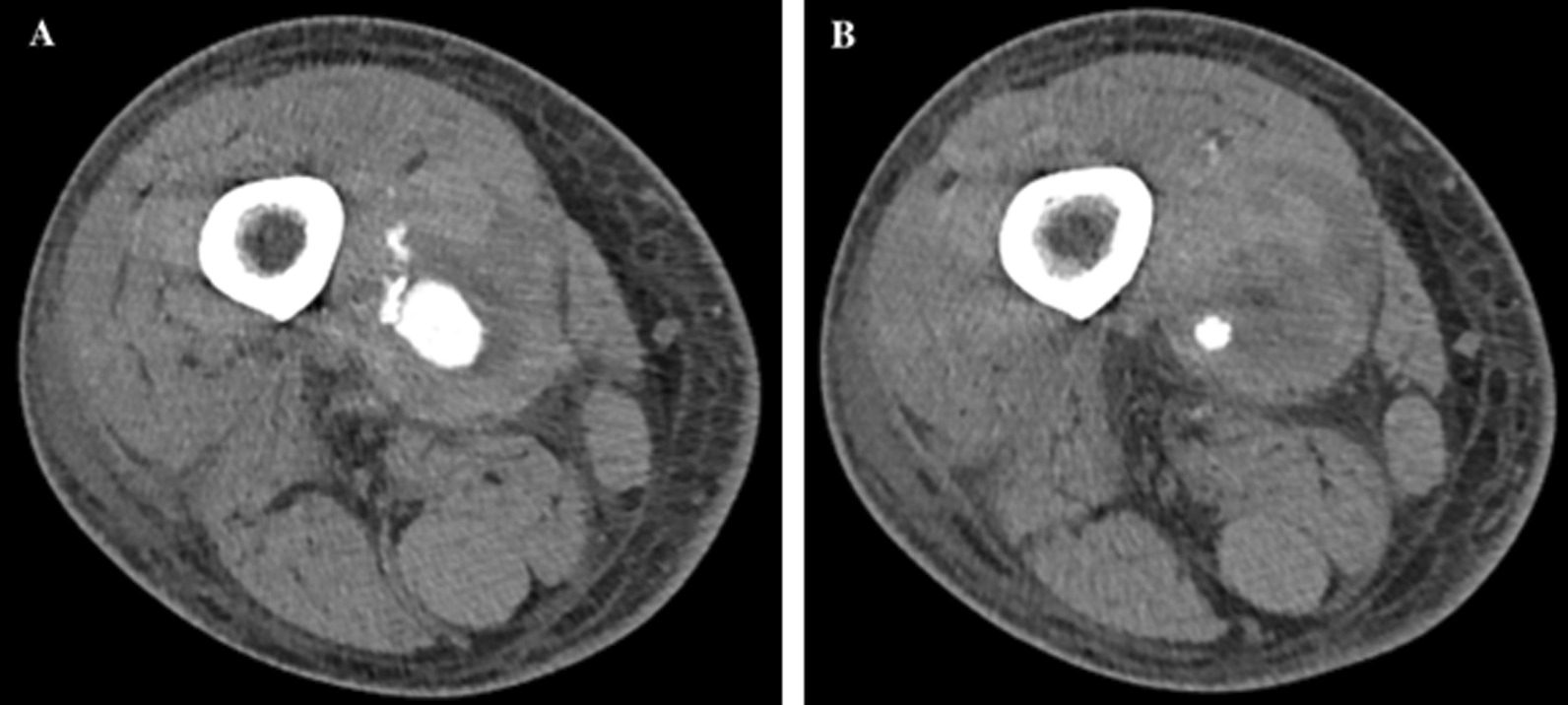
CT angiogram of right leg. CT angiogram with contrast of the right lower limb showing enhancement of the mycotic aneurysm (**A**) and hematoma formation (**B**)

Laboratory analysis revealed a leukocyte count of 13.9 × 109/L (4.5–11 × 109/L), hemoglobin of 10.2 g/dL (13.5–17.5 g/dL), prothrombin time 17.1 s (11.4–14.4s), international normalized ratio (INR) 1.4 (0.9–1.1s), aspartate transaminase level of 48 U/L, alanine aminotransferase level of 18 U/L, and total bilirubin of 2.1 mg/dL. Doppler ultrasound of the affected area revealed focal dilation of the right superficial femoral artery with “to and fro” type flow, concerning for a pseudoaneurysm. Subsequent computerized tomography angiogram confirmed pseudoaneurysm of the right superficial femoral artery, measuring 2 cm × 2 cm × 3 cm with active hemorrhage into a large right medial thigh hematoma measuring 10 cm × 8 cm × 24 cm (Fig. 1). Blood cultures grew gram-negative bacilli, but multiplex molecular blood culture panel failed to detect any of the organisms included in the panel.

**Fig. 2:**
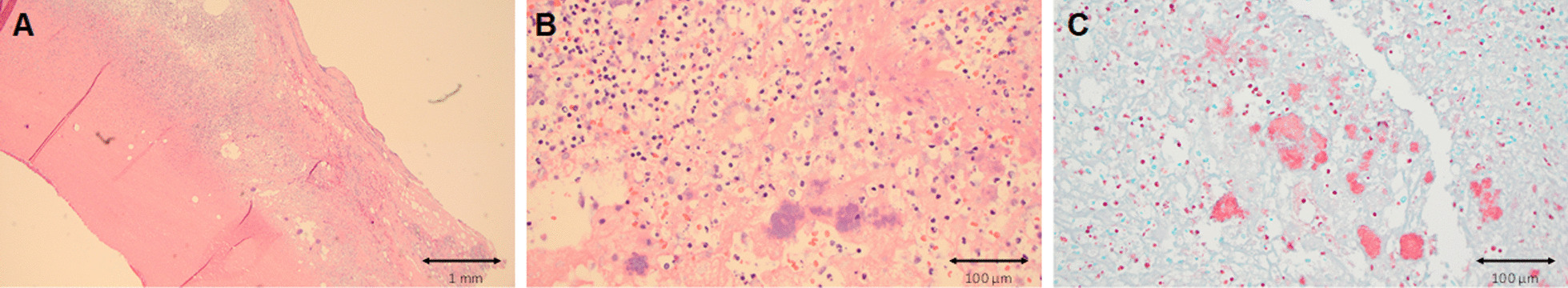
Histology of resected aneurysmal tissue. Hematoxylin and eosin stain of the excised aneurysmal tissue revealed bacterial colonies in the vascular wall tissue at (40× magnification) (**A**). The bacterial colonies are better seen at a higher power (400× magnification) (**B**). Gram stain of the tissue shows gram-negative bacteria (**C**)

He was transferred to our tertiary care hospital for further management. He underwent emergent open resection of the right femoral mycotic aneurysm and interposition bypass with the left greater saphenous vein. Intraoperatively, the site was noted to be grossly infected with multiple pockets of abscess. Pus cultures obtained during surgery grew *P. multocida* which was susceptible to ampicillin, ceftriaxone, and trimethoprim-sulfamethoxazole, and antibiotics were changed to ceftriaxone 2 g every 24 h. Blood cultures from the first hospital ultimately grew the same organism. Magnetic resonance imaging (MRI) of spine revealed small bilateral soft tissue abscesses in the medial paraspinal muscles at L3 without evidence of osteomyelitis. Transthoracic echocardiogram showed no findings suggestive of infective endocarditis. Because of a high index of clinical suspicion for infective endocarditis, tansesophageal echocardiogram was obtained and showed severe tricuspid regurgitation without evidence of endocarditis. Histologic examination of the aneurysm revealed acute inflammation with organizing thrombus and calcification. Histologic pictures in Fig. 2 were taken using Olympus BX46 microscopy with mounted Olympus UC90 camera and cellSens Entry software. The pictures have resolution of 3840 × 2160 with 96 dpi and no manual enhancement or editing of the pictures was done. Bacterial colonies were present on hematoxylin and eosin staining of the tissue (Fig. 2B) and gram stain confirmed that gram-negative bacteria were present within the excised aneurysm (Fig. 2C). Therefore, the diagnosis of *P. multocida* bacteremia with right femoral mycotic aneurysm, and right thigh and L3 lumbar paraspinal abscesses was made. The patient was discharged to a rehabilitation facility where he completed 6 weeks of intravenous ceftriaxone therapy. Patient had weekly complete blood count and complete metabolic panel and treatment adherence/tolerability was ascertained by regular telephone calls. Patient had in person infectious disease clinic visits every 3 weeks. His surgical wound healed.

## Discussion and conclusions

Mycotic aneurysms of peripheral arteries are rare and can arise due to bacteremia, direct bacterial inoculation, septic emboli, or spread from a nearby infection sites such as osteomyelitis [3]. Timely diagnosis is essential to prevent complications from untreated mycotic aneurysm including rupture, hemorrhage, distant embolization, and loss of limb viability [4]. However, diagnosis can be difficult because symptoms are often nonspecific, commonly including fever, pain, and inflammation at the site of infection. Patients with preexisting aneurysms, atherosclerosis, recent infection, and impaired immunity are at higher risk for development of mycotic aneurysm [4]. Numerous bacteria and fungi have been implicated in the formation of mycotic aneurysms, however Staphylococcus species and Salmonella species remain the most common causative organisms [3, 5]. Our patient had an extensive work up for endovascular infection including blood cultures, computerized tomography, and echocardiograms. Management of our patient included both surgical intervention and targeted antibiotic therapy.

Mycotic aneurysm can be diagnosed with imaging, intraoperative findings, and culture of the affected tissue [5]. Blood culture may also be useful in patients with suspected disseminated infection. In this case, both tissue and blood cultures confirmed *P. multocida* infection. The multiplex molecular blood culture panel did not detect any organisms as *P. multocida* is not included in the panel. Histologic examination of the tissue supports the diagnosis when bacteria are present, as seen in Fig. 2, demonstrating gram-negative bacteria within the aneurysmal tissue.

Only 10 cases of mycotic aneurysms due to *P. multocida* have been described in the literature (Table 1). In 8 of these 10 cases, the mycotic aneurysm involved the abdominal and thoracic aorta, and the 2 remaining cases had mycotic aneurysm cerebral vessels. To our knowledge, this is the first case of mycotic aneurysm of the superficial femoral artery due to *P. multocida*. Like our patient, nearly all previously reported patients had a known history of contact with a dog or cat (9/10), and infection was often associated with bacteremia (8/10). Our patient also had involvement of the paraspinal muscles, likely due to hematogenous spread.

**Table 1:** Reported cases of endovascular infections by *Pasteurella* complicated by aneurysm

Ref.	Age/ sex	Risk factor	Comorbid Conditions	Aneurysm location	Associated *Pasteurella* infection	Antimicrobial	Surgical management	Outcome
[11]	61, M	No bites/scratches, dogs and cats in home	Rheumatoid arthritis	Abdominal aorta	Left elbow and right first middle carpal septic joints, bacteremia	Penicillin G	Open surgical repair	Died during surgical repair
[12]	61, F	Cat bite	Not described	Thoracic aorta	None described	Not described	Open surgical repair	Not described
[13]	17, M	No known animal contact	Renal insufficiency	Cerebral aneurysm	Mitral valve endocarditis, bacteremia	Ampicillin	None	Died from CVA
[6]	54, M	Dog lick on psoriatic lesions	Laënnec’s cirrhosis, psoriasis inversa	Thoracic/abdominal aorta	Bacteremia	Amoxicillin, gentamicin	EVAR 1 year following	Alive 2 years following diagnosis
[7]	64, M	Cats in home	Heavy alcohol abuse	Abdominal aorta	Right leg cellulitis	Cefotaxime	Open surgical repair	Alive 1 year after surgery
[8]	68, M	Cat bite	Heavy alcohol abuse	Abdominal aorta	Right thumb cellulitis, bacteremia	Piperacillin-tazobactam	Open surgical repair	Died on day 13 post-surgery from septic shock
[14]	69, F	Cat bite	Not described	Abdominal aorta	Bacteremia	Penicillin G	EVAR followed by open surgical repair due to reinfection	Alive 8 months following surgery
[15]	61, M	Dog lick	Myelodysplastic Syndrome	Abdominal aorta and aortic arch	Bacteremia	Ampicillin	Open surgical repair	Alive 1 year following surgery
[16]	611, M	Dog bite	Not described	Descending thoracic aorta aneurysm	Bacteremia	Not described	Open surgical repair	Alive 18 months following surgery
[17]	57, F	Dog lick and scratch	Cigarette smoking	Cerebral aneurysm	Mitral valve endocarditis, Bacteremia	Penicillin G	Open surgical clipping	Alive 4 weeks following surgery
Our case	62, M	Dog scratch	Cirrhosis, Cigarette Smoking	Superficial femoral artery	Bacteremia, paraspinal abscesses	Ceftriaxone	Open surgical repair	Under treatment

Interestingly, a history of cirrhosis and/or heavy alcohol consumption was mentioned in three of the previously described cases and in our case [6–8]. A positive correlation between cirrhosis and *P. multocida* bacteremia has been shown in several studies. Chatelier et al. described 119 patients with *P. multocida* bacteremia and found that 24.4% of the patients had preexisting cirrhosis [2]. Similarly, Raffi et al. described 95 patients with *P. multocida* bacteremia, 34% of which were cirrhotic [9]. It is hypothesized that impaired reticuloendothelial function and portosystemic shunting allow for bacterial spread in these patients [2]. Simultaneously, cirrhosis and heavy alcohol consumption have been associated with the formation of mycotic aneurysms due to impaired immunity [10]. Our patient also had the additional risk factor of heavy cigarette smoking that likely contributed to underlying atherosclerosis, allowing bacterial seeding in the damaged vessel.

Treatment of mycotic aneurysms requires both surgical intervention and antibiotic therapy. Surgical intervention involves debridement of the infected tissue and revascularization when possible [4]. Our patient underwent en bloc resection of the aneurysm and adjacent femoral artery with autologous reconstruction using greater saphenous vein harvested from the contralateral leg. While there are no standardized guidelines for the duration of antibiotic treatment, the American Heart Association recommends 6 weeks of post-operative treatment for patients with peripheral mycotic aneurysms or extended up to 6 months for cases complicated by gross purulence or multidrug-resistant organisms [3]. *P. multocida* is typically sensitive to numerous antibiotics including penicillin, aminopenicillins, third and fourth generation cephalosporins, carbapenems, fluoroquinolones, doxycycline, and trimethoprim-sulfamethoxazole. Our patient is being treated with intravenous ceftriaxone.

This case highlights the importance of detailed history taking and prompt intervention in patients with mycotic aneurysms. Our patient had numerous risk factors that contributed to his presentation, including cirrhosis, cigarette smoking, and significant animal exposure. While animal scratches are somewhat common in pet owners, complications from bacterial invasion can occur and a complete history is essential for identifying risk factors.

## Conclusions

*P. multocida* is an exceptionally rare cause of mycotic aneurysm. Management should include both intravenous antibiotics and prompt surgical intervention to mitigate possible complications including aneurysm rupture, hemorrhage, and limb loss.

## Data Availability

The datasets used and/or analyzed during the current study are available from the corresponding author on reasonable request.

## Abbreviations

dL: Deciliters
INR: International normalized ratio

## References

[1] Wilson BA, Ho M (2013). *Pasteurella multocida*: from zoonosis to cellular microbiology. Clin Microbiol Rev.

[2] Chatelier E, Mahieu R, Hamel JF, Chenouard R, Lozac’h P, Salle A (2020). Pasteurella bacteraemia: impact of comorbidities on outcome, based on a case series and literature review. Int J Infect Dis.

[3] Wilson WR, Bower TC, Creager MA, Amin-Hanjani S, O’Gara PT, Lockhart PB (2016). Vascular graft infections, mycotic aneurysms, and endovascular infections: a scientific statement from the American Heart Association. Circulation.

[4] Deipolyi AR, Rho J, Khademhosseini A, Oklu R (2016). Diagnosis and management of mycotic aneurysms. Clin Imaging.

[5] Huang YK, Chen CL, Lu MS, Tsai FC, Lin PL, Wu CH (2014). Clinical, microbiologic, and outcome analysis of mycotic aortic aneurysm: the role of endovascular repair. Surg Infect (Larchmt).

[6] Balestra B (2000). Mycotic aneurysms of the aorta caused by infection with *Pasteurella multocida*. Clin Infect Dis.

[7] Koelemay MJ (2009). *Pasteurella multocida* infection, a rare cause of mycotic abdominal aortic aneurysm. J Vasc Surg.

[8] Cho DD, Berliner Y, Carr D (2016). Deadly case of *Pasteurella multocida* aortitis and mycotic aneurysm following a cat bite. World J Clin Cases.

[9] Raffi F, Barrier J, Baron D, Drugeon HB, Nicolas F, Courtieu AL (1987). *Pasteurella multocida* bacteremia: report of thirteen cases over twelve years and review of the literature. Scand J Infect Dis.

[10] Yoneda K, Shiraki K, Tanaka J, Beppu T, Fuke H, Yamamoto N (2007). Cervical mycotic aneurysm in a patient with alcoholic cirrhosis. Intern Med.

[11] Pestana OA (1974). Mycotic aneurysm and osteomyelitis secondary to infection with Pasteurella multocida. Am J Clin Pathol..

[12] Goldstein RW, Goodhart GL, Moore JE (1986). Pasteurella multocida infection after animal bites. N Engl J Med..

[13] Thamlikitkul V, Sangruchi T (1990). Pasteurella multocida infective endocarditis: a case report. J Med Assoc Thai..

[14] Shalan A, Wilson N, Poels J, Ikponmwosa A, Cavanagh S (2017). The Case of the Neighbour's Cat Causing a Symptomatic (Mycotic) Aortic Aneurysm and an Infected Endograft. EJVES Short Rep..

[15] Kano Y, Takamatsu A, Honda H (2020). Mycotic aneurysm due to Pasteurella multocida. QJM..

[16] Jeng EI, Acosta G, Martin TD, Upchurch GR (2020). Pasteurella Multiocida infection resulting in a descending thoracic aorta mycotic pseudoaneurysm. J Card Surg..

[17] Kollu VS, Archibald L, Edwards M, Janelle JW, Hong KW, Kalyatanda G (2021). Pasteurella Cerebral Mycotic Aneurysm: A Case Report and Review of the Literature. Cureus..

